# Changes in the use patterns of bDMARDs in patients with rheumatic diseases over the past 13 years

**DOI:** 10.1038/s41598-021-94504-x

**Published:** 2021-07-23

**Authors:** Carlos Sánchez-Piedra, Diana Sueiro-Delgado, Javier García-González, Inmaculada Ros-Vilamajo, Agueda Prior-Español, Manuel José Moreno-Ramos, Blanca Garcia-Magallon, Jerusalen Calvo-Gutiérrez, Yanira Perez-Vera, Raquel Martín-Domenech, Dolores Ruiz-Montesino, Paloma Vela-Casasempere, Lorena Expósito, Fernando Sánchez-Alonso, Enrique González-Davila, Federico Díaz-González

**Affiliations:** 1 Unidad de Investigación SER, Madrid, Spain; 2 Servicio de Reumatología, Complejo Hospitalario de Ourense, Ourense, Spain; 3 Servicio de Reumatología, Hospital Universitario Doce de Octubre, Madrid, Spain; 4 Servicio de Reumatología, Hospital Son Llatzer, Palma de Mallorca, Spain; 5 Servicio de Reumatología, Hospital Universitari Germans Trias i Pujol, Barcelona, Spain; 6 Servicio de Reumatología, Hospital Universitario Virgen Arrixaca, Murcia, Spain; 7 Servicio de Reumatología, Hospital San Jorge, Huesca, Spain; 8 Servicio de Reumatología, Hospital Reina Sofía, Cordoba, Spain; 9 Servicio de Reumatología, Hospital Universitario de Gran Canaria Doctor Negrín, Las Palmas, Spain; 10 Servicio de Reumatología, Hospital General de Elda, Alicante, Spain; 11 Servicio de Reumatología, Hospital Universitario Virgen Macarena, Seville, Spain; 12 Servicio de Reumatología, Hospital General Universitario Alicante, Alicante, Spain; 13 Servicio de Reumatología, Hospital Universitario de Canarias, Calle Ofra s/n 38320, La Laguna, Santa Cruz de Tenerife, Spain; 14 Departamento de Matemáticas, Estadística e Investigación Operativa, Universidad de La Laguna, Tenerife, Spain; 15 Departamento de Medicina Interna, Dermatología y Cirugía, Dermatología y Psiquiatría, Universidad de La Laguna, Tenerife, Spain

**Keywords:** Rheumatology, Rheumatic diseases, Medical research

## Abstract

The better understanding of the safety of biologic DMARDs (bDMARDs), as well as the emergence of new bDMARDs against different therapeutic targets and biosimilars have likely influenced the use patterns of these compounds over time. The aim of this study is to assess changes in demographic characteristics, disease activity and treatment patterns in patients with rheumatoid arthritis (RA), psoriatic arthritis (PsA), or ankylosing spondylitis (AS) who started a first- or second-line biologic between 2007 and mid-2020. Patients diagnosed with RA, PsA or AS included in the BIOBADASER registry from January 2007 to July 2020 were included. According to the start date of a first- or second-line biologic therapy, patients were stratified into four time periods: 2007–2009; 2010–2013; 2014–2017; 2018–2020 and analyzed cross-sectionally in each period. Demographic and clinical variables, as well as the type of biologic used, were assessed. Generalized linear models were applied to study the evolution of the variables of interest over time periods, the diagnosis, and the interactions between them. A total of 4543 patients initiated a first biologic during the entire time frame of the study. Over the four time periods, disease evolution at the time of biologic initiation (*p* < 0.001), disease activity (*p* < 0.001), retention rate (*p* < 0.001) and the use of tumor necrosis factor inhibitors as a first-line treatment (*p* < 0.001) showed a significant tendency to decrease. Conversely, comorbidities, as assessed by the Charlson index (*p* < 0.001), and the percentage of patients using bDMARDs in monotherapy (*p* < 0.001), and corticosteroids (*p* < 0.001) tended to increase over time. Over the entire period of the study's analysis, 3289 patients started a second biologic. The following trends were observed: decreased DAS28 at switching (*p* < 0.001), lower retention rates (*p* = 0.004), and incremental changes to the therapeutic target between the first and second biologic (*p* < 0.001). From 2007 until now rheumatic patients who started a biologic were older, exhibited less clinical activity, presented more comorbidities, and switched to a different biologic more frequently and earlier.

## Introduction

Over the last 2 decades, management of the most common chronic inflammatory rheumatic diseases involving joints has undergone a revolution since the arrival of biologic disease-modifying antirheumatic drugs (bDMARDs)^1^. Initially, information on the safety of biological products was limited, particularly regarding their long-term use. In addition, other factors, such as the heterogeneity of patients, the presence of comorbidities and risk factors, as well as treatment adherence, also slowed the widespread use of these products^2^. Although it is evident that bDMARDs have improved the clinical outcomes of patients with rheumatic diseases, these treatments are not without significant costs. Biological agents are expensive and the rheumatic diseases that require their use affect more than 2% of the population^3,4^. Therefore, even the richest societies cannot support the indiscriminate use of bDMARDs in all of the patients who require them^5,6^.

It is reasonable to think that the arrival of both new families of bDMARDs with different therapeutic targets^7,8^ and more affordable versions of the original bDMARDs (biosimilars)^9,10^ has contributed to the increased use of these products. However, the clinical experience gained with these compounds has been particularly decisive in the increasing use of biologics, resulting in a change in the profile of rheumatic patients treated with bDMARDs over the last 2 decades. Nevertheless, despite all these changes, few studies have assessed how the use patterns of these compounds in rheumatic patients have changed since bDMARDs have become available. Most studies that have analyzed changes over time in the use patterns of bDMARDs have focused almost exclusively on rheumatoid arthritis (RA) patients^11,12^. However, studies analyzing these temporary changes in the use of bDMARDs in psoriatic arthritis (PsA)^13^ or ankylosing spondylitis (AS) remain very scarce.

It is likely that a better understanding of the safety and effectiveness of biologics, as well as the appearance of new therapeutic targets and biosimilars, has influenced the manner in which patients with chronic musculoskeletal inflammatory disease are treated. The objective of this analysis was to compare the baseline characteristics of patients with RA, PsA or AS, including demographic and disease characteristics, who underwent a first- and second-line biological therapy from 2007 to mid-2020.

## Methods

### Study design and setting: BIOBADASER III

BIOBADASER (*biobadaser.ser.es*) is a Spanish multicenter observational registry aimed at assessing safety in patients with rheumatic diseases who started treatment with any bDMARD or targeted synthetic DMARD. It was established in 2000 and has been comprehensively described in a previous report^14^. BIOBADASER III is the third stage of this registry, a new version developed in December 2015^15^, which added to its objectives a systematic assessment of effectiveness using commonly accepted indexes. The BIOBADASER registry is promoted by the Spanish Society of Rheumatology (SER) and is supported by the Spanish Agency of Drugs and Medical Devices (AEMPS) as well as by several pharmaceutical companies. To assess consistency and quality, strict measures are implemented. Every year the full database is monitored online; additionally, a random sample of patients is annually selected and audited in situ at all 28 participating centers. The recruitment of new patients is dynamic and remains open indefinitely. Further details about the design and operation of the BIOBADASER III registry, such as the complete protocol and relevant documentation, are available at the BIOBADASER website (biobadaser.ser.es).

Patients diagnosed with RA, PsA and/or AS included in the BIOBADASER database who started either a first or second-line biologic between January 2007 and July 2020 were analyzed. Four independent cross-sectional analyses were conducted during the following time periods: 2007–2009; 2010–2013; 2014–2017; 2018–2020. For each period, demographic and clinical variables, the type of biologic used as well as biologic retention rates were assessed in patients who started a first- or second-line biologic. All parameters were analyzed cross-sectionally, except for the retention rate, which was analyzed prospectively as the percentage of patients who maintained that particular biologic therapy 1 year after its initiation.

### Participants

Included in this analysis were all BIOBADASER registry patients diagnosed with RA, PsA or AS according to the criteria used by their treating rheumatologist and who were prescribed a bDMARD, either naïve (first-line) or after failing another drug of these classes (second-line), from 1 January 2007 to 20 July 2020.

The number of participating centers varied across the different periods. In BIOBADASER Phase II (2007–2015) all patients who started biologic treatment owing to a rheumatic disease in the 14 participating hospitals were included. During BIOBADASER Phase III (2015–2020), the number of centers ranged from 25 to 35 depending on recruitment capacity and data quality.

### Variables

Patients were classified according to the year they started the biological treatment (first-line) or the year they switched to a second biological treatment (second-line). Four time periods were arbitrarily established for the analysis: first, from 2007 to 2009; second, from 2010 to 2013; third, from 2014 to 2017; and fourth, from 2018 to 2020.

This analysis included the following variables: (1) demographics (sex and age); (2) clinical characteristics, such as disease activity by DAS28 in RA and PsA patients, and comorbidities based on pathologies or the Charlson comorbidity index^16^; (3) treatment data, including type of biologic, date of initiation and discontinuation, the use of synthetic DMARDs and glucocorticoids (GCs). The retention rate at the end of the first year of follow-up was also analyzed. Compared with the previous version, BIOBADASER III collects disease activity indices of patients with RA, PsA, and AS prospectively and also includes the assessment of comorbidities using the Charlson index, although only at the baseline visit. Although BIOBADASER III collects BASDAI in patients with AS, this has been required only from 2014; thus, AS activity was not evaluated in this study.

All procedures and materials complied with the principles of the Declaration of Helsinki and with Spanish regulations on data protection and research. The project was approved by the Ethics Review Committee of the Hospital Universitario Clinic Barcelona, which acted as the reference committee (approval code FER-ADA-2015-01). Informed consent was obtained from all participants.

### Statistical analysis

The sample was described in terms of the distribution of the descriptive variables through measures of central tendency. The quantitative variables were expressed as mean and standard deviations, except for the disease evolution time to the first biologic, which was expressed as median and interquartile ranges due to its strong positive asymmetry. For qualitative variables, the frequencies and percentages were calculated, the latter rounded to one decimal place. Chi-Square tests were used to test independence. Generalized linear models were applied to study the evolution of the variables of interest over the relevant time periods, the diagnosis, and the interaction between them. The study of the interaction enables us to analyze the possible variations in the outcomes analyzed when the patients are in a specific time and diagnosis category with respect to the reference category, which, in our study, was patients with RA in the period 2007–2010, denoted as (2007–2010) and RA. Identity was used as a link for quantitative variables and logit link for qualitative variables. For disease evolution time, a logarithmic transformation was initially applied to normalize and homogenize the variances. Treatment duration was analyzed, taking into account the time of treatment until discontinuation, and including only those patients who had discontinued treatment. A Tukey DHS (Honestly Significant Difference) procedure was performed to determine the homogeneity of the quantitative variables across the different time periods. Statistical analyses were performed by using STATA software (version 13.1).

## Results

### First-line biological treatment

From 1 January 2007 to 20 July 2020, a total of 4543 patients starting bDMARDs were registered in the BIOBADASER database. The first period (from 2007 to 2009) comprised 1522 patients; the second (from 2010 to 2013), 786 patients; the third (from 2014 to 2017), 886 patients; and finally fourth period (from 2018 to July 2020), 1349 patients were analyzed.

Table 1 shows the demographic characteristics of patients who began a first-line biologic treatment by time periods. Most participants were female (58.6%) and had been diagnosed with RA (48.3%). Regarding the mean age at the beginning of the first biologic, although it increased from 51.0-years-old in the first time period to 52.1-years-old in the fourth, this variable does not reach statistical significance (*p* = 0.075). When patients were assessed as a whole, the median disease evolution time at the beginning of the first biologic fell from 5.5 in the first period to 3.4 years (*p* < 0.001) in the fourth (Table 2). In terms of pathology, a significant downward trend was similarly detected in all three pathologies: RA patients decreased from 5.7 to 4.3 years (*p* = 0.001), PsA patients from 5.0 to 3.0 years (*p* < 0.001), and AS patients from 5.9 to 2.5 years (*p* = 0.001). An analysis of homogeneous subsets revealed a tendency to use biologics in the first indication in patients with a significantly shorter disease progression in the second period than in the first period for all three diseases analyzed. In RA and PsA patients, disease activity at the beginning of the first biologic, as assessed by DAS28, was consistently higher, from 5.3 to 4.7 (*p* < 0.001) and 4.9 to 4.2 (*p* < 0.001), during the first (2007–2009) time period versus the fourth (2018–2020), respectively (Table 2).

**Table 1:** Diagnosis and sociodemographic features at the beginning of first-line biologics treatment by time periods.

Variable	2007–2009	2010–2013	2014–2017	2018–2020	p	Total
Number of participating centers*	14	13	27	31		38
Number of patients included	1522	786	886	1349		4543
**Diagnosis, n (%)**					< 0.001	
RA	870 (57.2)	390 (49.6)	328 (37.0)	605 (44.8)		2193 (48.3)
PsA	318 (20.9)	194 (24.7)	280 (31.6)	414 (30.7)		1206 (26.6)
AS	334 (21.9)	202 (25.7)	278 (31.4)	330 (24.5)		1144 (25.2)
**Demographic characteristics**						
Female gender, n (%)	933 (61.3)	462 (58.8)	456 (51.5)	809 (60.0)	0.104	2660 (58.6)
Age at the beginning of first biologic, mean ± SD	51.0 ± 14.2	50.9 ± 14.0	50.5 ± 13.3	52.1 ± 13.2	0.075	51.2 ± 13.7

*Centers that included at least one patient in this analysis.

**Table 2:** Clinical features and use of treatments at the beginning of first-line biologics treatment by time periods.

Variable	2007–2009	2010–2013	2014–2017	2018–2020	*p*	Homogeneous subsets**	Total
**Clinical features**							
Disease evolution time to first biologics in years, median (P_25_;P_75_)	5.5 (1.9; 11.9)	4.1 (1.5; 10.3)	3.7 (1.2; 8.9)	3.4 (1.1; 8.3)	< 0.001	1 2 3 4	4.2 (1.4;10.0)
RA	5.7 (2.2; 12.1)	4.5 (1.8; 10.3)	5.4 (2.2; 10.8)	4.3 (1.7; 9.3)	< 0.001	1 2 3 4	5.0 (2.0; 10.5)
PsA	5.0 (1.7; 9.6)	3.5 (1.4; 9.1)	3.0 (1.0; 7.2)	3.0 (1.0; 7.0)	< 0.001	1 2 3 4	3.4 (1.2; 8.0)
AS	5.9 (1.4; 14.6)	4.2 (0.8; 12.6)	2.6 (0.8; 9.1)	2.5 (0.7; 8.6)	< 0.001	1 2 3 4	3.6 (0.9; 11.8)
*DAS28 at baseline, mean ± SD*							
RA	5.3 ± 1.3	4.8 ± 1.4	4.8 ± 1.1	4.7 ± 1.3	< 0.001	1 2 3 4	4.9 ± 1.3
PsA	4.9 ± 1.3	4.8 ± 1.5	4.1 ± 1.3	4.2 ± 1.2	< 0.001	1 2 3 4	4.4 ± 1.3
**Treatments**							
Use of TNF inhibitors as first-line treatment, n (%)	1441 (94.7)	669 (85.1)	660 (74.5)	989 (73.3)	< 0.001	1 2 3 4	3759 (82.7)
RA	791 (90.9)	277 (71.0)	195 (59.5)	459 (75.9)	< 0.001	1 2 3 4	1722 (78.5)
PsA	317 (99.7)	191 (98.5)	210 (75.0)	255 (61.6)	< 0.001	1 2 3 4	973 (80.7)
AS	333 (99.7)	201 (99.5)	255 (91.7)	275 (83.3)	< 0.001	1 2 3 4	1064 (93.0)
Monotherapy	444 (29.2)	284 (36.1)	338 (38.2)	576 (42.7)	< 0.001	1 2 3 4	1642 (36.1)
*Concomitant therapy, n (%)*							
Methotrexate	830 (54.5)	362 (46.1)	329 (37.1)	522 (38.7)	< 0.001	1 2 3 4	2043 (45.0)
Leflunomide	232 (15.2)	135 (17.2)	176 (19.9)	207 (15.3)	0.014	1 2 3 4	750 (16.5)
Others	157 (10.3)	68 (8.7)	117 (13.2)	128 (9.5)	0.010	1 2 3 4	470 (10.3)
Use of corticosteroids	701 (46.1)	323 (41.1)	334 (37.7)	542 (40.2)	< 0.001	1 2 3 4	1900 (41.8)
Retention rate at end of first year of follow-up, n (%)	1138 (74.8)	571 (72.7)	599 (67.6)	678 (50.3)	< 0.001	1 2 3 4	2986 (65.7)
RA	587 (67.5)	260 (66.7)	196 (59.8)	312 (52.6)	< 0.001	1 2 3 4	1355 (61.8)
PsA	266 (83.7)	152 (78.4)	202 (72.1)	200 (48.3)	< 0.001	1 2 3 4	820 (68.0)
AS	285 (85.3)	159 (78.7)	201 (72.3)	166 (50.3)	0.001	1 2 3 4	811 (70.9)
**Comorbidities**							
Charlson Index, mean ± SD	1.6 ± 0.9	1.6 ± 0.9	1.9 ± 1.4	2.0 ± 1.3	< 0.001	1 2 3 4	1.8 ± 1.2
Cancer, n (%)	29 (1.9)	14 (1.8)	33 (3.7)	64 (4.7)	< 0.001	1 2 3 4	140 (3.1)
Lymphoma, n (%)	4 (0.3)	1 (0.1)	1 (0.1)	4 (0.3)	0.905	1 2 3 4	10 (0.2)
Ischemic heart disease, n (%)	37 (2.4)	18 (2.3)	25 (2.8)	36 (2.7)	0.574	1 2 3 4	116 (2.6)
Diabetes, n (%)	120 (7.9)	46 (5.9)	52 (5.9)	112 (8.3)	0.773	1 2 3 4	330 (7.3)
Heart failure, n (%)	23 (1.5)	10 (1.3)	9 (1.0)	15 (1.1)	0.285	1 2 3 4	57 (1.3)
Hypercholesterolemia, n (%)	226 (14.9)	114 (14.5)	225 (25.4)	325 (24.1)	< 0.001	1 2 3 4	890 (19.6)
Arterial hypertension, n (%)	343 (22.5)	159 (20.2)	210 (23.7)	315 (23.4)	0.373	1 2 3 4	1027 (22.6)
Osteoporosis, n (%)	179 (11.8)	70 (8.9)	69 (7.8)	115 (8.5)	0.002	1 2 3 4	433 (9.5)
Smoker, n (%)	266 (17.5)	128 (16.3)	225 (25.4)	296 (21.9)	< 0.001	1 2 3 4	915 (20.1)

*Centers that included at least one patient in this analysis.

**Numbers in the homogeneous subsets column represent the four consecutive periods of time. Lines join time periods in which differences were not statistically significant.

Regarding treatment (Table 2), the use of TNFi as a first-line treatment option was significantly lower in patients as a whole, from 94.7% in the first period to 73.3% in the fourth (*p* < 0.001). This trend was also confirmed when patients were analyzed by pathologies. With regards to concomitant therapy, the use of biologics in monotherapy was higher over the different analyzed time periods: from 29.2 to 42.7% (*p* < 0.001) of patients. In addition, the use of corticosteroids also decreased significantly (*p* < 0.001), with a clear downward trend from the first to the third period, 46.1–37.7%, respectively, albeit with a mild increment reaching 40.2% in the fourth period. With respect to the retention rate of the first biologic during the first year of follow-up (Table 2), it decreased in patients overall from 74.8% in the first period to 50.3% in the fourth (*p* < 0.001). This trend was also confirmed when patients were analyzed by pathologies. With respect to comorbidities (Table 2), the Charlson index showed a significant tendency to increase over the time periods of the study (*p* < 0.001). Cancer antecedents (*p* < 0.001), hypercholesterolemia (*p* < 0.001), osteoporosis (*p* = 0.008) and smoking status (*p* < 0.001) were more frequently recorded in patients who started bDMARDs treatment during the 2018–2020 period with respect to those who did so during the 2007–2009 period.

**Figure 1:**
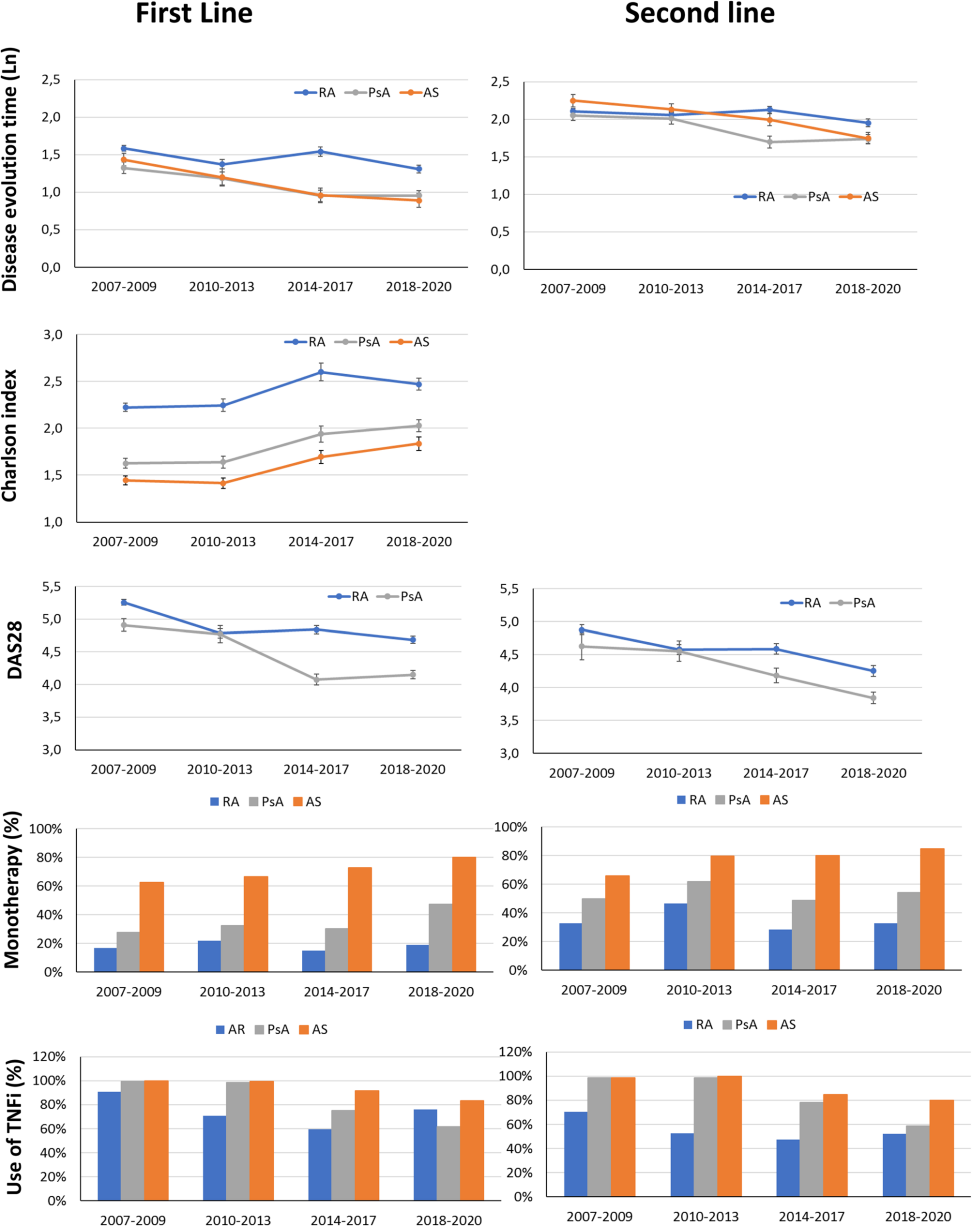
Changes over time periods in terms of disease evolution time, Charlson comorbidity index, DAS28, use of TNFi and monotherapy according to diagnosis. First- and second-line biological treatment trends are shown.

Figure 1 shows the changes over time in disease evolution time with respect to the first biologic, Charlson index, DAS28, use of TNF inhibitors and monotherapy according to the diagnosis and study period.

In an additional analysis using a generalized linear model (see Supplementary Table 1 online), the interaction between diagnosis (reference category: RA) and period (reference: 2007–2010) was evaluated in the group of patients that initiated a biologic. Over the study period, first-line biologics were used in patients with shorter disease evolution time, with less disease activity, with more comorbidities and with a tendency to use therapeutic targets other than TNF. When analyzed by diagnosis, patients with PsA or AS who initiated the first biologic had shorter disease evolution times, less disease activity (patients with PsA), and less comorbidities, but more frequently underwent anti-TNF in monotherapy than did RA patients. When the interaction between the period and diagnosis was analyzed using the same references (initial period and RA diagnosis), patients with PsA, especially from 2014 onwards, exhibited a shorter disease evolution time, less disease activity, and less use of anti-TNF, preferably in monotherapy over the study time period with respect to RA. In this model, no interaction between the study time period and diagnosis was found for comorbidities, as assessed by the Charlson index.

The distribution of bDMARDs varied across time periods (see Supplementary Table 2 and Supplementary Fig. 1 online). Biosimilars first became available on the Spanish market in 2016. Since that time, the percentage of patients with RA, PsA or AE who started treatment with a biosimilar as a first-line therapy has gradually increased to 65% by 2020 (see Supplementary Fig. 2A online).

### Second-line biological treatment

During the time period of this study, BIOBADASER registered a total of 3289 patients who started treatment with a second biologic: 827 patients during the first period (from 2007 to 2009); 884 patients in the second (2010–2013); 733 patients in the third (2014–2017); and 845 patients in the fourth (2018–2020). Most patients were female (62.9%) and had been diagnosed with RA (55.4%) (Table 3).

**Table 3:** Diagnosis and sociodemographic features at the beginning of a second biologic (second-line treatment) by time period.

Variable	2007–2009	2010–2013	2014–2017	2018–2020	*p*	Total
Number of participating centers*	14	14	26	29		36
Number of patients included	827	884	733	845		3289
**Diagnosis, n (%)**					< 0.001	
RA	549 (66.4)	548 (62.0)	370 (50.5)	356 (42.1)		1823 (55.4)
PsA	154 (18.6)	152 (17.2)	182 (24.8)	289 (32.2)		777 (23.6)
AS	124 (15.0)	184 (20.8)	181 (24.7)	200 (23.7)		689 (21.0)
**Demographic characteristics**						
Female gender, n (%)	539 (65.2)	594 (67.2)	443 (60.4)	491 (58.1)	< 0.001	2067 (62.9)
Age at the beginning of second biologic, mean (SD)	51.0 ± 14.2	50.9 ± 14.0	50.5 ± 13.3	52.1 ± 13.2	0.417	53.6 ± 13.3

*Centers that included at least one patient in this analysis.

Table 4 shows the clinical and treatment characteristics, by time period, of those patients who began a second-line biologic treatment. In patients overall, disease evolution from the time a second biologic was introduced decreased significantly from 11.6 years in the first period to 9.6 years in the fourth (*p* < 0.001). As was the case with first-line biologic treatment, biologics were prescribed more frequently in monotherapy to patients who initiated second-line biologics over the different time periods analyzed (41.1–52.5%; *p* < 0.001). By pathologies, this trend was also noted in PsA patients, from 10.5 to 8.7 (*p* < 0.001), and in AS patients, from 13.6 to 9.6 (*p* = 0.003), but not in RA patients (*p* = 0.147). Regarding disease activity, as assessed by DAS28, both RA and PsA patients at the beginning of a second biologic decreased significantly from 4.6 to 4.0 (*p* < 0.001) and 4.2 to 3.3 (*p* < 0.001) in the first and fourth periods, respectively. Over the four time periods analyzed, the first switching showed a clear tendency to change the initial therapeutic target to the detriment of TNF inhibitor use in the three pathologies studied. In contrast to what occurred with the first-line treatment, the use of bDMARDs in monotherapy decreased significantly (*p* < 0.001) throughout the 4 time periods. In addition, the use of TNF inhibitors also consistently decreased throughout the 4 time periods: from 79.8% in the first, to 54.1% (*p* < 0.001) in the fourth. The first year’s retention rate regarding the second biologic showed a significant trend to decrease, both in terms of patients overall, from 55.3%, in the first period to 46.15% in the fourth (*p* < 0.001), and by pathologies (Table 4).

**Table 4:** Clinical features and use of treatments at the beginning of a second biologic (second-line treatment) by time period.

Variable	2007–2009	2010–2013	2014–2017	2018–2020	*p*	Homogeneous subsets**	Total
**Clinical features**							
Disease evolution time when second biologic was used in years, median (P_25_;P_75_)	9.2 (4.8; 15.5)	9.1 (4.3; 15.9)	8.3 (3.8; 15.0)	7.2 (3.4; 12.5)	< 0.001	1 2 3 4	8.5 (4.1; 14.8)
RA	9.3 (4.8; 15.5)	8.9 (4.3; 15.7)	9.4 (4.8; 15.7)	8.8 (4.1; 13.5)	0.184	1 2 3 4	9.1 (4.5; 15.1)
PsA	8.4 (4.6; 13.6)	8.7 (4.0; 15.0)	6.8 (2.5; 12.7)	6.5 (3.3; 11.3)	0.006	1 2 3 4	7.3 (3.4; 13.4)
AS	10.9 (5.1; 19.8)	9.7 (4.7; 17.4)	8.5 (3.8; 17.0)	6.5 (2.5; 12.0)	0.002	1 2 3 4	8.4 (3.9; 16.4)
*DAS28 at baseline, mean ± SD*							
RA	4.9 ± 1.6	4.6 ± 1.5	4.6 ± 1.4	4.3 ± 1.5	< 0.001	1 2 3 4	4.6 ± 1.5
PsA	4.6 ± 1.9	4.6 ± 1.5	4.2 ± 1.4	3.8 ± 1.4	< 0.001	1 2 3 4	4.2 ± 1.5
**Use of treatments**							
Change of therapeutic target between 1st and 2nd biologic, n (%)	141 (17.1)	174 (19.7)	225 (30.7)	232 (38.2)	< 0.001	1 2 3 4	863 (26.2)
Use of TNF inhibitors as a second-line treatment, n (%)	660 (79.8)	621 (70.8)	471 (64.3)	514 (60.8)	< 0.001	1 2 3 4	2266 (68.9)
RA	386 (70.3)	287 (52.4)	176 (47.6)	185 (52.0)	< 0.001	1 2 3 4	1034 (56.7)
PsA	152 (98.7)	150 (98.7)	142 (78.0)	169 (58.5)	< 0.001	1 2 3 4	613 (78.9)
AS	122 (98.4)	184 (100.0)	153 (84.5)	160 (80.0)	< 0.001	1 2 3 4	619 (89.8)
Monotherapy	340 (41.1)	496 (56.1)	340 (46.4)	444 (52.5)	< 0.001	1 2 3 4	1620 (49.3)
*Concomitant therapy, n (%)*							
Methotrexate	354 (42.8)	289 (32.7)	246 (33.6)	263 (31.1)	< 0.001	1 2 3 4	1152 (35.0)
Leflunomide	141 (17.0)	100 (11.3)	110 (15.0)	114 (13.5)	0.006	1 2 3 4	465 (14.1)
Others	84 (10.2)	51 (5.8)	73 (10.0)	57 (6.7)	0.001	1 2 3 4	265 (8.1)
Use of corticosteroids	329 (39.8)	264 (29.9)	285 (38.9)	285 (33.7)	0.214	1 2 3 4	1163 (35.4)

*Centers that included at least one patient in this analysis.

**The homogeneous subsets are represented by lines joining the time periods in which differences were not statistically significant.

Overall, except for specific variations, the patterns observed in both the first- and second-line use of biologicals maintained this same trend (Fig. 1). In the generalized linear model, using the same references (2007–2009 as the time period and RA as the diagnostic measure), a second-line biologic was consistently used across the study periods in those patients with less active disease. In addition, there was a trend to preferably use therapeutic targets other than TNF, albeit not in monotherapy or in cases with a shorter disease evolution time. When analyzed by diagnosis, patients with PsA and AS who used a second-line biologic were more often administered anti-TNF in monotherapy than were RA patients. When the interaction between the time period and diagnosis was analyzed, we found that the tendency to administer a second-line biologic correlated with shorter disease evolution times and, in monotherapy, in AS patients over the respective time periods. (see Supplementary Table 3 online).

Similar to what was observed with the first-line treatment, the use of biosimilars as a second-line therapy gradually increased in patients with rheumatic diseases since the arrived of these drugs on the market in 2016, reaching 46% in 2020 (see Supplementary Fig. 2B online).

## Discussion

The most important findings of this work can be summarized as follows: (1) From 2007 to 2020, the demographic characteristics, clinical activity and the therapeutic target of bDMARDs chosen for patients with chronic inflammatory arthropathies, both as first- and second-line treatments, have changed; (2) Over this period of time, the profile of a patient who started a first-line biologic tended to have a shorter disease duration, lower clinical disease activity, and more comorbidities; (3) With respect to bDMARDs, the use of TNFi as a first-line treatment has decreased, with a clear temporal trend towards the use of bDMARDs in monotherapy in RA, PsA and AS; and (4) during the time frame analyzed, the persistent use of first-line biologics decreased significantly, the first bDMARD switching occurred with lower disease activity, both in RA and PsA patients, and TNFi, as a second therapeutic option, was less frequently used in all three pathologies analyzed.

bDMARDs have transformed the treatment of RA, PsA and AS, improving outcomes for patients who do not tolerate or properly respond to conventional DMARDs. However, in the absence of evidence supporting relevant differences in the effectiveness and/or safety among the available bDMARDs for rheumatic diseases, most international recommendations leave their selection primarily up to the physician's discretion^17–21^. This lack of guidance on the most appropriate approach for prescribing bDMARDs, as well as improvements in the general knowledge about their long-term safety, an increased number of available therapeutic targets and the greater affordability of biosimilars, are all factors that may have influenced the use patterns of these compounds over time. During the period of time analyzed in our study, a trend towards shorter disease evolutions, lower clinical disease activity and more comorbidities was observed in those RA and PsA patients who had begun a first-line biologic. Apart from disease activity, which was not assessed, this same tendency was observed in AS patients. These results are consistent with the trends initially described in previous studies on RA in different countries. In the United Kingdom, Hyrich et al. assessed changes in disease characteristics in patients who had started TNFi for RA between 2001 and 2008, finding that there was a significant trend towards earlier use of TNFi in those with less severe disease^11^. Norwegian and Swedish studies, analyzing equivalent periods of time, reported similar results in RA patients^2,22^. In both studies, the earlier use of TNFi in RA correlated with improved outcomes^11,22^. An Italian real-life clinical study covering the period from 1999 to 2015, which included a limited number of RA patients, showed an increasing tendency for earlier introduction of bDMARDs during the disease course, even in those with moderate disease activity and less severity^12^. With regards to PsA patients, a previous study reported a trend to lower disease duration in PsA patients starting bDMARDs during the period 1999–2008^2^. A recently published Nordic population-based study on PsA patients who initiated bDMARDs from 2006 to 2017 showed that in recent years these compounds have been prescribed when disease activity is lower compared to previous years. In this study, changes over time in disease evolution and comorbidities at the start of bDMARDs treatment were not studied^13^. As with RA and PsA patients, we found a trend towards shorter disease evolution time in AS patients, both when a first or second biologic was started. As far as we know, no previous study has analyzed changes in disease duration and activity over time in AS patients treated with bDMARDs. Regarding the presence of comorbidities vis-à-vis the initiation of a first-line bDMARD, we observed a trend towards a higher Charlson index and higher incidences of cancer history, hypercholesterolemia, osteoporosis, and smoking status when analyzing the patient cohorts as a whole. This indicates that over the last decade, at least in our series, the use of bDMARDs has become less restrictive in regards to the previous health status of rheumatic patients. To the best of our knowledge, no study has analyzed changes over time in terms of the comorbidity profiles of rheumatic patients who initiate biologics. We believe that this trend may reflect an increased familiarity and better knowledge of the long-term safety profiles of these compounds by physicians. In our study, TNFi as a first-line bDMARDs therapy option showed a significant trend over time towards lower use, when analyzing the entire population or by pathology. The percentage of patients using TNFi as a first-line treatment declined sharply from 2007 to 2020 in PsA and AS patients, probably due to the arrival during that time of new bDMARDs with different therapeutic targets (IL-6, IL-17 or IL-12/23). However, during the last time period analyzed, 2018–2020, this trend slowed in RA patients, showing an upturn. This similarly can be explained by TNFi biosimilars entering the marketplace at very competitive prices. Biosimilars for rheumatic diseases have been available in Europe since 2016 and have reduced costs for some drugs by as much as 75%, though in many cases far less. Most current clinical practice guidelines include biosimilars in their therapeutic strategies^17–19^, although some reluctance on using these compounds in routine clinical practice persists^23,24^. However, the disparity in the penetration of biosimilars in different markets does not yet allow for an assessment of their real effects on therapeutic strategies.

International guidelines for RA and PsA generally recommend using bDMARDs in combination with conventional DMARDs^17^. However, recent evidence from routine clinical practice suggests that in these two pathologies, the retention rates for bDMARDs do not depend on their use in monotherapy or in combination, except for TNFi in RA, in which drug survival was significantly lower in monotherapy^25–27^. Throughout our study population, the use of bDMARDs in monotherapy increased progressively throughout the periods of time analyzed. Indeed, it showed that in daily clinical practice this treatment strategy has gradually adopted bDMARDs as a first-line treatment. A previous study analyzing 9764 RA patients from 2006 to 2010 found that one-third of patients received first-line TNFi in monotherapy, which is consistent with our data during a similar time frame (36.13%). In PsA patients studied from 2004 to 2012, the percentage that initiated treatment with TNFi in monotherapy was also around one-third^28^. Improved knowledge of the clinical response to bDMARDs acquired in real-life clinical settings, in addition to poor tolerance of methotrexate (MTX)^29,30^, may be reasons that have contributed to the increasing use of bDMARDs in monotherapy for patients with rheumatic diseases.

In our study, a tendency to reduce the persistence time of a first biologic, switching it for other bDMARDs in patients with less disease activity, was observed both in RA and PsA. A study in the US that included a large number of RA patients from two insurance programs, the data showed that the annual rate of bDMARD switching increased significantly between 2000 and 2015. The reasons for switching, including disease activity, were not assessed in this study^31^. However, another US study using a real-world database from 2001 to 2009, found that the threshold of disease activity for switching biologic treatments decreased over time in RA patients^32^. During a similar time frame, but in Europe, RA patients discontinuing TNFi showed a temporary trend towards decreased disease activity at the time of discontinuation^33^.

Cycling versus swapping strategies for bDMARDs has been a controversial issue^34^. However, most observational studies, including well-designed prospective randomised analyses, have demonstrated the superiority of swapping over cycling as the best approach for managing TNFi non-responding RA patients, whereas only a few studies have reported any comparable effectiveness^35–38^. In AS patients, a study including patients that initiated TNFi from 2009 to 2013 confirmed that while many patients cycled through TNFi medications, IL-17 inhibitors were not available during the relevant time period. Thus, it is not possible to know whether switching to IL-17 inhibitors would have impacted the observed treatment patterns^39^. These findings are consistent with our own results: an increased use of bDMARDs (non-TNFi's) as a second-line treatment for RA and PsA patients over time. In RA, we observed an increased use of TNFi during the last time period analyzed with respect to the previous one, probably due to the arrival of TNFi biosimilars at very competitive prices beginning in 2016. In AS patients, the use of TNFi as a second-line treatment occurred only until 2015, when IL-17 inhibitors entered the marketplace. The introduction of the treat-to-target (T2T) approach may also have influenced these changes in the pattern of bDMARD use over the past few years^40–42^. In particular, shorter disease evolution times to initiate bDMARDs, and lower clinical activity when switching are two patterns that could be associated with the adoption of this strategy.

This study has both strengths and limitations. Our study analyzed data from a relevant number of patients included in an annually monitored national registry that has been active since 2000. Nonetheless, some limitations are obvious. Our evaluation of the activity of patients with PsA was done using DAS28 instead of more specific activity indices, such as DAPSA. In this work, disease activity in patients with PsA was assessed using DAS28 instead of more specific indices, such as DAPSA^43^. DAS28 was originally developed as an activity index for RA, and although it has been used for the assessment of PsA in previous studies^44,45^, the use of DAS28 in PsA may not be accurate in patients with PsA presenting with joint patterns that are not polyarticular or RA-like^46^. Although for the last 2 years BIOBADASER is collecting disease activity using both DAPSA and DAS28 in PsA patients, the rationale for using DAS28 to assess PsA activity in our study was that no other activity index was available for PsA patients in BIOBADASER from 2007 to 2018. In addition, the activity of patients with AS could not be studied because of the absence of BASDAI data prior to 2014. The decision to evaluate changes in the pattern of bDMARDs use over arbitrary time periods should not affect the objective of our analysis or the trends identified over time. Finally, we found differences in the proportion of patients according to diagnosis across the time periods studied. In order to limit the effect of these differences on our findings, we analyzed key variables (disease duration to first/second biologics in years, DAS28, retention rate at end of first year of follow-up, use of TNF inhibitors as a first/second-line treatment) for each condition and used generalized linear models to study changes in the variables of interest over the time periods, after considering the interaction between them and adjusting for the diagnosis.

## Conclusions

There have been changes in the use patterns of bDMARDs as both first- and second-line treatments over the last 13 years. Patient profiles have evolved and are now characterized by shorter disease evolution times and lower disease activity. The tendency to use bDMARDs as a first-line treatment in patients with more comorbidities was observed. Treatment strategies involving bDMARDs have also changed: the utilization of TNFi has decreased globally, and now the use of monotherapy strategies as first and/or second-line treatments is much more common.

## Supplementary Information

Supplementary Figure 1.

Supplementary Figure 2.

Supplementary Table 1.

Supplementary Table 2.

Supplementary Table 3.

## Data Availability

The data that support the findings of this study are available from Spanish Society of Rheumatology but restrictions apply to the availability of these data, which were used under license for the current study, and so are not publicly available. Data are however available from the authors upon reasonable request and with permission of Spanish Society of Rheumatology.
