# Supplementary material for: Changes in the use patterns of bDMARDs in patients with rheumatic diseases over the past 13 years
Source: Sci Rep. 2021 Jul 23;11:15051. doi: 10.1038/s41598-021-94504-x (PMC8302725; doi:10.1038/s41598-021-94504-x)
Supplement: Supplementary file 1 — Supplementary Figure 1. [file 41598_2021_94504_MOESM1_ESM.docx]

**Supplementary Figure 1.**


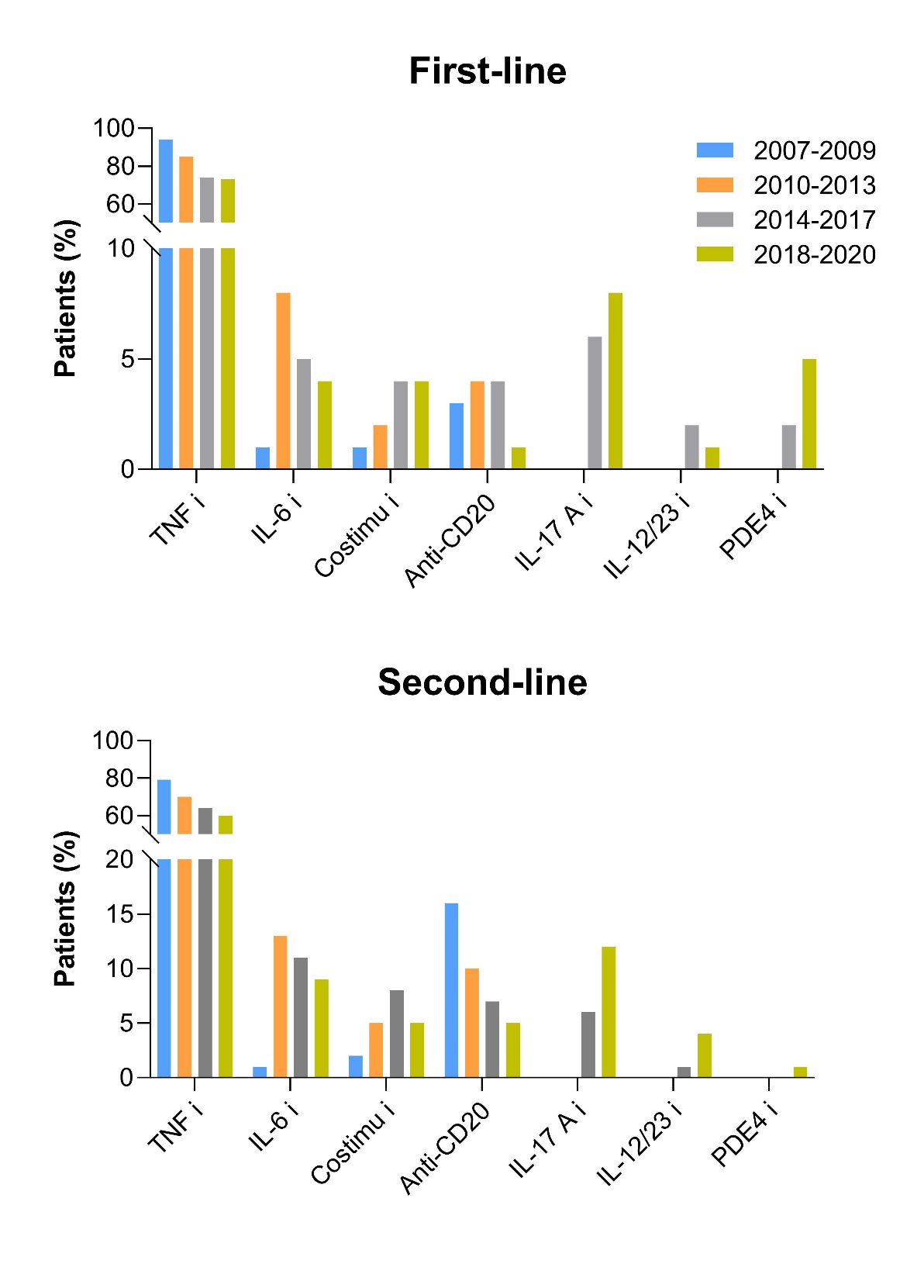


Figure legend: Distribution of bDMARD families used as first and second line of treatment over different time periods. Data are expressed as percentage of patients treated with each group of biologics in each period.

bDMARD, biologic disease-modifying anti-rheumatic drugs; IL-6 i, interleukin-6 inhibitors; TNF i, tumor necrosis factor inhibitors; Costimu i, costimulation inhibitors; IL-17A i : interleukin-17A inhibitors; IL-12/23 i, interleukin-12/23 inhibitor.
