# Supplementary material for: Changes in the use patterns of bDMARDs in patients with rheumatic diseases over the past 13 years
Source: Sci Rep. 2021 Jul 23;11:15051. doi: 10.1038/s41598-021-94504-x (PMC8302725; doi:10.1038/s41598-021-94504-x)
Supplement: Supplementary file 2 — Supplementary Figure 2. [file 41598_2021_94504_MOESM2_ESM.docx]

**Supplementary Figure 2.**

**A**


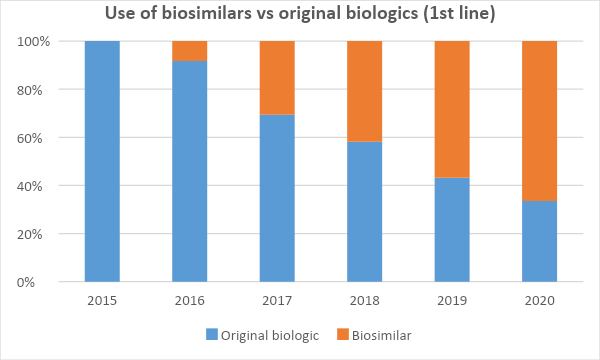


**B**


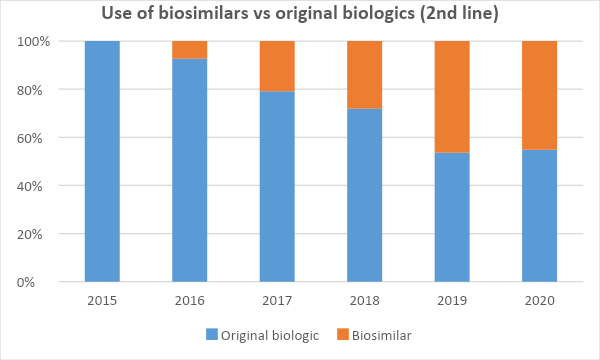


Figure legend: Evolution in the use of biosimilars since 2015. First- and second-line trends in the use of biological treatments. A) Impact of biosimilars on the use of bDMARDs as a first-line treatment. B) Impact of biosimilars on the use of bDMARDs as a second-line treatment.
