# Supplementary material for: Changes in the use patterns of bDMARDs in patients with rheumatic diseases over the past 13 years
Source: Sci Rep. 2021 Jul 23;11:15051. doi: 10.1038/s41598-021-94504-x (PMC8302725; doi:10.1038/s41598-021-94504-x)
Supplement: Supplementary file 3 — Supplementary Table 1. [file 41598_2021_94504_MOESM3_ESM.docx]

**Supplementary Table 1.** Results from generalized linear models for the outcomes: Disease evolution time to first biologic, Charlson index, DAS28, use of TNF inhibitors as first-line treatment and monotherapy. Results by period, diagnosis, and interaction. First-line treatment.

|  | Disease evolution time^**^ | Das28 | Charlson index | Use of TNF inhibitors | Monotherapy |
| --- | --- | --- | --- | --- | --- |
| Period (ref. 2007-2010) |  |  |  |  |  |
| 2010-2013 | -0.21 (0.08); 0.010 | -0.47 (0.08); <0.001 | 0.02 (0.08); 0.779 | -1.41 (0.16); <0.001 | 0.34 (0.15); 0.026 |
| 2014-2017 | -0.04 (0.09); 0.627 | -0.42 (0.09); <0.001 | 0.38 (0.08); <0.001 | -1.92 (0.16); <0.001 | -0.11 (0.18); 0.521 |
| 2018-2020 | -0.28 (0.07); <0.001 | -0.57 (0.07); <0.001 | 0.24 (0.07); <0.001 | -1.16 (0.15); <0.001 | 0.15 (0.14); 0.271 |
| Diagnostic (ref. RA) |  |  |  |  |  |
| PsA | -0.26 (0.09); 0.003 | -0.35 (0.11); 0.001 | -0.60 (0.08); <0.001 | 3.45 (1.01); 0.001 | 0.64 (0.15); <0.001 |
| As | -0.15 (0.09); 0.083 | - | -0.78 (0.08); <0.001 | 3.50 (1.01); 0.001 | 2.13 (0.14); <0.001 |
| Period _*_ Diagnostic (ref. (2007-2010) & RA) |  |  |  |  |  |
| (2010-2013) & PsA | 0.07 (0.15); 0.628 | 0.34 (0.17); 0.045 | -0.01 (0.14); 0.951 | -0.20 (1.17); 0.886 | -0.11 (0.25); 0.660 |
| (2010-2013) & AS | -0.02 (0.15); 0.870 | - | -0.05 (0.14); 0.707 | 0.90 (1.43); 0.527 | -0.16 (0.24); 0.495 |
| (2014-2017) & PsA | -0.33 (0.14); 0.019 | -0.42 (0.15); 0.006 | -0.06 (0.14); 0.640 | -2.74 (1.02); 0.007 | 0.24 (0.25); 0.335 |
| (2014-2017) & AS | -0.43 (0.14); 0.002 | - | -0.13 (0.13); 0.338 | -1.48 (1.04); 0.154 | 0.58 (0.25); 0.020 |
| (2018-2020) & PsA | -0.09 (0.12); 0.461 | -0.19 (0.14); 0.164 | 0.16 (0.12); 0.181 | -4.13 (1.02); <0.001 | 0.71 (0.21); 0.002 |
| (2018-2020) & AS | -0.27 (0.13); 0.036 | - | 0.14 (0.12); 0.232 | -3.04 (1.02); 0.003 | 0.71 (0.22); 0.002 |

The data show the model coefficients (standard error); p-value.

Link function: identity for disease evolution time to first-line biologics, Charlson index and DAS28, and logit for use of TNF inhibitors as a first-line treatment and monotherapy.

^**^Logarithmic transformation.
