# Supplementary material for: Changes in the use patterns of bDMARDs in patients with rheumatic diseases over the past 13 years
Source: Sci Rep. 2021 Jul 23;11:15051. doi: 10.1038/s41598-021-94504-x (PMC8302725; doi:10.1038/s41598-021-94504-x)
Supplement: Supplementary file 4 — Supplementary Table 2. [file 41598_2021_94504_MOESM4_ESM.docx]

**Supplementary Table 2.** Distribution of bDMARDs used as first- and second-line of treatment across time periods.

|  | 2007-2009 |  | 2010-2013 |  | 2014-2017 |  | 2018-2020 |  |
| --- | --- | --- | --- | --- | --- | --- | --- | --- |
|  | first-line | second-line | first-line | second-line | first-line | second-line | first-line | second-line |
| Etanercept | 527 (34.6) | 308 (37.2) | 171 (21.8) | 238 (26.9) | 220 (24.8) | 112 (15.3) | 406 (30.1) | 150 (17.8) |
| Infliximab | 361 (23.7) | 92 (11.1) | 106 (13.5) | 58 (6.6) | 78 (8.8) | 68 (9.2) | 43 (3.2) | 38 (4.5) |
| Adalimumab | 551 (36.2) | 260 (31.4) | 317 (40.3) | 241 (27.3) | 152 (17.2) | 120 (16.4) | 396 (29.3) | 199 (23.6) |
| Anakinra | 0 (-) | 1 (0.1) | 1 (0.1) | 1 (0.1) | 3 (0.3) | 0 (-) | 0 (-) | 0 (-) |
| Rituximab | 47 (3.1) | 132 (16.0) | 32 (4.1) | 92 (10.4) | 39 (4.4) | 53 (7.2) | 25 (1.8) | 45 (5.3) |
| Abatacept | 15 (1.0) | 22 (2.7) | 19 (2.4) | 50 (5.7) | 42 (4.7) | 59 (8.0) | 62 (4.6) | 45 (5.3) |
| Tocilizumab | 19 (1.2) | 12 (1.5) | 65 (8.3) | 120 (13.6) | 49 (5.5) | 83 (11.3) | 27 (2.0) | 47 (5.6) |
| Golimumab | 0 (-) | 0 (-) | 51 (6.5) | 66 (7.5) | 118 (13.3) | 112 (15.3) | 80 (5.9) | 82 (9.7) |
| Certolizumab | 2 (0.1) | 0 (-) | 24 (3.1) | 18 (2.0) | 92 (10.4) | 59 (8.0) | 68 (5.0) | 45 (5.3) |
| Ustekinumab | 0 (-) | 0 (-) | 0 (-) | 0 (-) | 21 (2.4) | 14 (1.9) | 15 (1.1) | 35 (4.1) |
| Apremilast | 0 (-) | 0 (-) | 0 (-) | 0 (-) | 18 (2.0) | 6 (0.8) | 79 (5.9) | 16 (1.9) |
| Secukinumab | 0 (-) | 0 (-) | 0 (-) | 0 (-) | 54 (6.1) | 47 (6.4) | 113 (8.4) | 92 (10.9) |
| Sarilumab | 0 (-) | 0 (-) | 0 (-) | 0 (-) | 0 (-) | 0 (-) | 31 (2.3) | 36 (4.3) |
| Ixekizumab | 0 (-) | 0 (-) | 0 (-) | 0 (-) | 0 (-) | 0 (-) | 4 (0.3) | 15 (1.8) |
| Total | 1522 | 827 | 786 | 884 | 886 | 733 | 1349 | 845 |

Data were expressed as absolute number and percentage of patent treated with each biologic in each period.
