# Supplementary material for: Changes in the use patterns of bDMARDs in patients with rheumatic diseases over the past 13 years
Source: Sci Rep. 2021 Jul 23;11:15051. doi: 10.1038/s41598-021-94504-x (PMC8302725; doi:10.1038/s41598-021-94504-x)
Supplement: Supplementary file 5 — Supplementary Table 3. [file 41598_2021_94504_MOESM5_ESM.docx]

**Supplementary table 3.** Results from generalized linear models for the outcomes: Disease evolution time to second biologic, DAS28, use of TNF inhibitors as a second treatment and monotherapy. Results by period, diagnosis and its interaction. Second-line treatment.

|  | Disease evolution time^**^ | DAS28 | Use of TNF inhibitors | Monotherapy |
| --- | --- | --- | --- | --- |
| Period (ref. 2007-2010) |  |  |  |  |
| 2010-2013 | -0.05 (0.06); 0.422 | -0.30 (0.11); 0.004 | -0.77 (0.13); <0.001 | 0.57 (0.13); <0.001 |
| 2014-2017 | 0.02 (0.06); 0.742 | -0.29 (0.11); 0.010 | -0.96 (0.14); <0.001 | -0.20 (0.15); 0.166 |
| 2018-2020 | -0.16 (0.07); 0.071 | -0.62 (0.11); <0.001 | -0.78 (0.14); <0.001 | 0.01 (0.14); 0.974 |
| Diagnosis (ref. RA) |  |  |  |  |
| PsA | -0.06 (0.09); 0.525 | -0.25 (0.18); 0.165 | 3.47 (0.72); <0.001 | 0.71 (0.19); <0.001 |
| As | 0.14 (0.09); 0.133 | - | 3.25 (0.72); <0.001 | 1.38 (0.21); <0.001 |
| Period _*_ Diagnosis (ref. (2007-2010) & RA) |  |  |  |  |
| (2010-2013) & PsA | 0.01 (0.12); 0.952 | 0.23 (0.25); 0.368 | 0.75 (1.02); 0.457 | -0.09 (0.26); 0.739 |
| (2010-2013) & AS | -0.07 (0.13); 0.576 | - | 1.87 (1.24); 0.131 | 0.14 (0.29); 0.632 |
| (2014-2017) & PsA | -0.38 (0.12); 0.002 | -0.15 (0.23); 0.512 | -2.10 (0.75); 0.005 | 0.16 (0.26); 0.546 |
| (2014-2017) & AS | -0.28 (0.13); 0.032 | - | -1.45 (0.76); 0.054 | 0.93 (0.30); 0.002 |
| (2018-2020) & PsA | -0.16 (0.12); 0.174 | -0.16 (0.22); 0.471 | -3.21 (0.73); <0.001 | 0.18 (0.25); 0.470 |
| (2018-2020) & AS | -0.35 (0.13); 0.007 | - | 0.86 (0.09); <0.001 | 1.07 (0.31); 0.001 |

The data show the model coefficients (standard error); p-value.

Link function: identity for disease evolution time to first biologic and “DAS28”, and logit for use of TNF inhibitors as a first treatment and monotherapy.

^**^Logarithmic transformation.
